# One-Lung Ventilation during Rigid Bronchoscopy Using a Single-Lumen Endotracheal Tube: A Descriptive, Retrospective Single-Center Study

**DOI:** 10.3390/jcm12062426

**Published:** 2023-03-21

**Authors:** Carolin Steinack, Helene Balmer, Silvia Ulrich, Thomas Gaisl, Daniel P. Franzen

**Affiliations:** 1 Department of Pulmonology, Interventional Lung Center, University Hospital Zurich, Raemistrasse 100, 8091 Zurich, Switzerland; 2 Department of Internal Medicine, Spital Uster, Brunnenstrasse 42, 8610 Uster, Switzerland

**Keywords:** rigid bronchoscopy, one-lung ventilation, single-lumen endotracheal tube

## Abstract

Using one-lung ventilation (OLV) through a single-lumen endotracheal tube (SLT) in the untreated lung during rigid bronchoscopy (RB) and jet ventilation, high oxygenation can be guaranteed, whilst procedures requiring thermal energy in the other lung are still able to be used. This pilot study aimed to examine the bronchoscopy-associated risks and feasibility of OLV using an SLT during RB in patients with malignant airway stenosis. All consecutive adult patients with endobronchial malignant lesions receiving OLV during RB from 1 January 2017 to 12 May 2021 were included. We assessed perioperative complications in 25 RBs requiring OLV. Bleeding grades 1, 2, and 3 complicated the procedure in two (8%), five (20%), and five (20%) patients, respectively. The median saturation of peripheral oxygen remained at 94% (*p* = 0.09), whilst the median oxygen supply did not increase significantly from 0 L/min to 2 L/min (*p* = 0.10) within three days after the bronchoscopy. The 30-day survival rate of the patients was 79.1% (95% CI 58.4–91.1%), all of whom reported an improvement in subjective well-being after the bronchoscopy. OLV using an SLT during RB could be a new treatment approach for endobronchial ablative procedures without increasing bronchoscopy-associated risks, allowing concurrent high-energy treatments.

## 1. Introduction

Patients with endobronchial lesions usually experience dyspnea, stridor, cough, hemoptysis, or poststenotic pneumonia, eventually, disabling essential chemotherapy for malignant lesions [1]. Endobronchial ablative treatments, including laser, argon plasma coagulation, cryotherapy, electrocautery, and airway stent placement have the potential to improve these symptoms [2,3]. Moreover, some procedures during bronchoscopy require the use of thermal energy, including argon plasma coagulation (APC), laser treatment, and electrocautery [2,4,5]. In these cases, oxygen levels of 100% would result in airway fire, when the ignition source encounters an oxidizer-rich environment [6]. Therefore, a fraction of inspired oxygen (FiO_2_) of ≤0.4 is recommended to minimize the risk of airway fire. In a healthy lung, FiO_2_ of ≤0.4 is generally well tolerated during bronchoscopy without complications [7]. However, ventilation-/perfusion mismatch during jet ventilation during rigid bronchoscopy (RB) is potentially impaired and some patients may have significant comorbidities. Thus, oxygen uptake in patients with pre-existing lung conditions is an issue when FiO_2_ must be decreased due to high-energy applications [8,9]. Consequently, one of the relative contraindications for high-thermal ablative treatments is patients requiring >0.4 FiO_2_ during bronchoscopy [2]. RB is an important technique applied in the respiratory and thoracic fields for diagnostic or therapeutic indications, including tumor debulking, foreign body removal, and the control of pulmonary hemorrhage [10]. Tumor debulking can be achieved by flexible or RB, the latter with a larger working channel enabling endobronchial access [10]. During RB, the patients are ventilated with high-flow jet ventilation (HFJV) applying sub-dead space tidal volumes using supra-physiological frequencies [11,12]. 

One-lung ventilation (OLV) is routinely used for thoracic anesthesia. Double-lumen tubes are the most popular and reliable choice for OLV in adult patients undergoing thoracic surgery [13]. During RB, double-lumen tubes cannot be administered, since their large outer diameter disables RB, while the inner diameter lacks sufficient space for endobronchial procedures. Therefore, patients with impaired oxygenation and a need for RB are intubated on the non-treated side using cuffed single-lumen tubes (SLTs) [14]. The use of SLTs is less popular; nonetheless, their use is effective for OLV [15,16]. The use of OLV can provide 1.0 FiO_2_ in the untreated lung side, whilst allowing the use of procedures requiring thermal energy in the other lung, without the risk of airway fire. 

This work aimed to provide data on the bronchoscopy-associated risks and feasibility of OLV during RB for patients with symptomatic malignant airway stenosis from a single center. 

## 2. Materials and Methods

### 2.1. Patient Selection and Study Design

All consecutive patients receiving OLV during RB at the University Hospital Zurich were enrolled in this descriptive, retrospective study from 1 January 2017 to 12 May 2021. Exclusion criteria were as follows: age < 18 years and lack of informed consent. All patient-related data, including demographic and clinical data, were obtained from electronic patient record files. These data included bronchoscopy reports, radiological and medical data, anesthesia protocols, and follow-up interviews. Cancer stage was classified according to the eighth edition of the American Joint Commission on Cancer (AJCC) TNM staging system for non-small cell lung cancer [17]. The study was approved by the Competent Ethics Committee of the Canton of Zurich (ID 2021-01380).

### 2.2. Bronchoscopy

All bronchoscopies were performed as in-patient procedures under general anesthesia and muscle relaxation using rigid bronchoscopes with an inner diameter between 8.5 and 16 mm (Storz, Tuttlingen, Germany) that were placed in the trachea. Subsequently, flexible bronchoscopes (190 series; Olympus, Tokyo, Japan) were inserted using a rigid bronchoscope. The respiratory frequency averaged between 100–150 breaths per min (BPM), and the applied pressure varied between 1.5 and 2.5 bar using the following formula: *p* = 1 + (0.01 × kg BW patient) atm [18,19]. The decision of OLV was made during RB, when thermal energy procedures were required and pulseoxymetric oxygen saturation (SpO_2_) declined below 88% during HFJV with FiO_2_ ≤ 0.4. After removal of the rigid bronchoscope and mask ventilation, an SLT with an inner diameter from 6.0 to 8.5 mm (Rüsch Bronchoflex, Willy Rüsch GmbH, Kernen, Germany) was inserted into the non-treated main bronchus with flexible bronchoscopy and cuffed for OLV, maintaining oxygen levels > 40%. The size of the SLT was chosen based on the body size. The cuff pressure was between 23 und 28 mmHg and was controlled at the beginning and during OLV. OLV was applied setting tidal volumes at 4–6 mL/kg bodyweight and positive end-expiratory pressure at 5–8 cm H_2_O. Subsequently, a rigid bronchoscope of size six or eight was introduced next to the SLT and placed in the distal trachea right in front of the orifice of the main treated bronchus, tracking the flexible bronchoscope for the ongoing procedure, while the non-treated bronchus with the cuffed SLT remained ventilated with >40% oxygen levels. HFJV was generally maintained using FiO_2_ < 0.4. Hence, thermal energy bronchial procedures, laser (Leonardo^®^, Biolitec, Vienna, Austria) with wavelengths of 980 nm and 1470 nm or argon plasma coagulation using ionized argon gas (VIO ^®^ 200D, APC 2, Erbe, Tübingen, Germany) from 20 to 40 W were used on the other side without a high risk of airway fire.

### 2.3. Outcomes

The general state and perioperative risk of the patients were evaluated using the American Society of Anesthesiology (ASA) score [20], and preoperative dyspnea was evaluated using the Modified Medical Research Council Dyspnea Scale (mMRC) [21]. Bronchoscopy-associated risks were analyzed based on perioperative adverse events. Averse events included hypoxemia, bleeding, fistula formation, bronchospasm, and hemodynamic instability [22]. Hypoxemia was defined as an SpO_2_ below 90% for at least 1 min [23,24]. Potential adverse events induced by HFJV included barotrauma. Regarding the bleeding grade, grade 1 bleeding required suctioning of blood for <1 min; grade 2 > 1 min, repeated wedging, or instillation of vasoactive substances or thrombin. Premature interruption of the procedure, balloon blocker insertion of <20 min, or selective intubation was classified as grade 3 bleeding. Grade 4 bleeding was defined as selective intubation or balloon blocker insertion for >20 min in addition to red blood cell transfusion, selective bronchial artery embolization, admission to the intensive care unit, surgical intervention, or resuscitation [25]. Relevant hemodynamic changes were defined as alterations in blood pressure >20 mmHg. Perioperative respiratory rates, oxygen supply, and SpO_2_ were observed within three days before and after bronchoscopy. We assessed pre-and postoperative symptoms within 30 days after the procedure. Alleviation of symptoms, subjective increase in well-being, and family-reported increase in patient activity noted in hospital records were interpreted as subjective improvement. The 30-day survival rate was chosen based on clinical research conducted in this field using a similar endpoint [24,26,27]. In addition, days of in-hospital stay and cases of re-bronchoscopy were reported.

### 2.4. Statistical Analysis

Quantitative data were presented as mean ± standard deviation (SD). Categorical variables were expressed as frequencies (*n*) and percentages (%). Data were analyzed using the SPSS software (version 26.0; IBM, New York, NY, USA) and STATA (v17, StataCorp LLC, College Station, TX, USA). Depending on the distribution of the data, a student’s *t*-test or a Mann–Whitney U test was used. For longitudinal comparisons within patients, a paired *t*-test or a Wilcoxon’s signed rank test was used to account for the dependency of the data. 

## 3. Results

### 3.1. Demographic and Clinical Data

**Figure 1:**
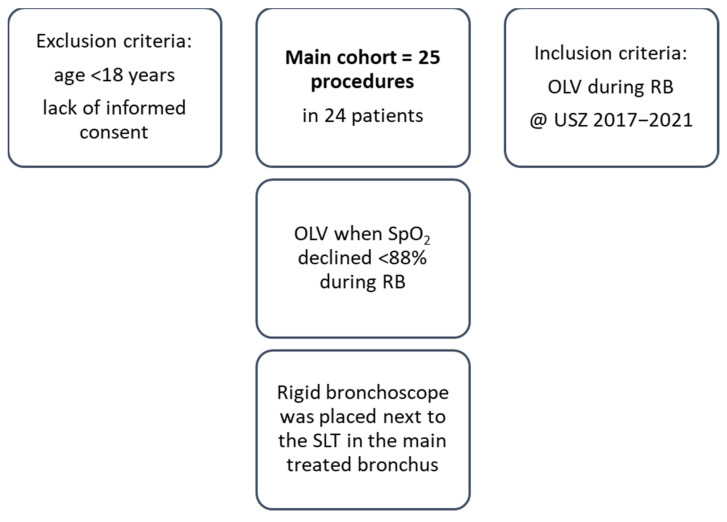
Study flow. Abbreviations: SpO_2_, saturation of peripheral oxygen; OLV, one-lung ventilation; RB, rigid bronchoscopy.

Totally, 25 procedures were performed in 24 patients (Figure 1). The majority were male (*n* = 16, 64%) and in palliative care (*n* = 19, 76%). Most of them had lung cancer (*n* = 21, 84%) frequently located in the perihilar region on the right side (*n* = 12, 48%). In total, 84% of the patients were active (20%) or former smokers (64%). Eight percent of the patients had prior lung resection, consisting of one wedge resection and one lobectomy. All patients had severe disease and were classified as ASA class 3 (72%) or 4 (28%) (Table 1). Preoperative cough was reported in 21 (84%) patients, whereas hemoptysis and non-bloody productive cough were present in 5 (20%) and 4 (16%) patients, respectively. A total of 96% of the patients had an mMRC dyspnea scale score of ≥1. 

### 3.2. Perioperative Data and Adverse Events

Perioperative data and adverse events were assessed within three days before and after bronchoscopy. Perioperative procedures are listed in Table 2 and perioperative medications used in Supplementary Materials Table S1. The mean duration of jet ventilation was 37 ± 23.7 min and the mean procedure time 80.6 ± 32.1 min.

Perioperative adverse events are shown in Table 3. The mean duration of hypoxemia (SpO_2_ < 90% for at least 1 min) was 12.5 ± 7.6 min with the lowest mean SpO_2_ of 81.7 ± 6.2%. In 88% (*n* = 22) of patients, OLV was performed after relevant hypoxemia had occurred. Subsequently, the SpO_2_ did not decrease below 90%. Only 12% (*n* = 3) of patients were previously scheduled for OLV during RB because of huge tumor obstruction of the main steam bronchus. Intraoperative hypoxemia was not recorded in these cases. Tumor-related bleeding grades 2 and 3 were the most common (20%) and were controllable by the bronchoscopist within the same operation. Bronchospasm occurred in one patient (4%). Blood pressure changes were related to hypoxemia in 69% (*n* = 11) of cases. Abnormal heart rhythm was noted in one (4%) patient with atrial fibrillation entailing two electro conversions due to desaturation before OLV has begun. Following this incident, the OLV was initiated with no further complications. A pre-existing broncho-mediastinal fistula was observed in one patient. Procedure-related bronchial fistulas or complications caused by the rigid tube were not recorded.

**Figure 2:**
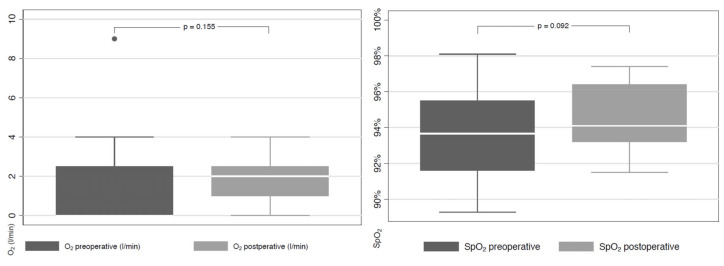
Oxygen saturation and supply before and after the intervention. Abbreviations: O_2_, oxygen supply; SpO_2_, saturation of peripheral oxygen.

The mean respiratory rates before and after the intervention were 20 ± 3 BPM and 20 ± 4 BPM, respectively. The median SpO_2_ remained at 94% (no statistical difference, *p* = 0.09), whilst the median oxygen supply did not increase significantly from 0 L/min to 2 L/min) (*p* = 0.10) within three days after bronchoscopy (Figure 2). Eleven patients had to increase their oxygen supply for one day after bronchoscopy, although they had no need for exogenous oxygen supply. Seven patients who already needed oxygen before bronchoscopy were able to reduce their oxygen supply. 

Although all patients had a high perioperative risk of ASA classes 3 and 4, including 15 (60%) patients with pre-existing lung comorbidities, we did not identify irreversible perioperative adverse events, sustained hypoxemia, or the need for a significant increase in oxygen supply. Once our treatment protocol was conducted and OLV with a cuffed SLT was placed on the non-treated side, SpO_2_ remained stable at >90%.

### 3.3. Postoperative Complications

**Figure 3:**
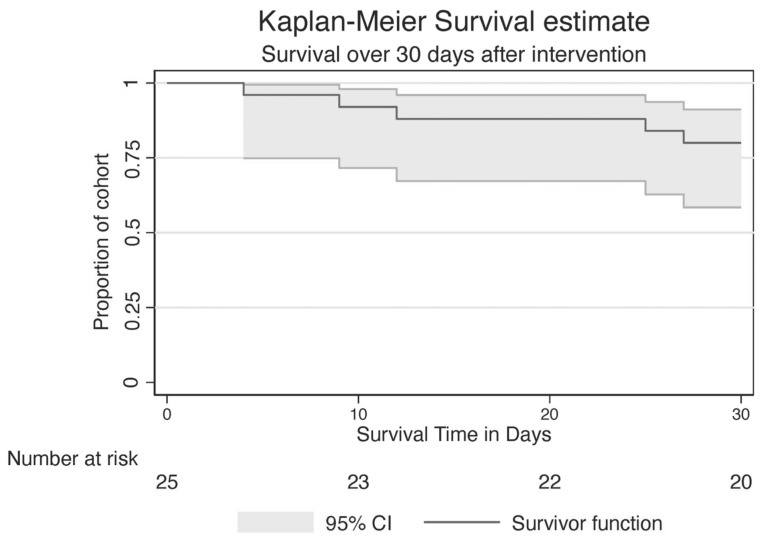
Kaplan–Meier survival estimate. Thirty-day survival was observed in 20 patients (79.1% [95% CI 58.4–91.1%]).

Thirty-day survival was achieved in 20 patients (79.1% [95% CI 58.4–91.1%]) (Figure 3), all of whom reported an improvement in subjective well-being after the bronchoscopy. Flexible re-bronchoscopy was necessary in 40% (*n* = 10) of patients (6 patients had routine control, 4 patients had stent restenosis). The mean length of in-hospital stay was 4 ± 4.9 days.

## 4. Discussion

OLV using a cuffed SLT during RB could be a new treatment approach for endobronchial ablative procedures to restore airway lumen patency without increasing bronchoscopy-associated risks, allowing concurrent high-energy treatments for patients with pre-existing lung conditions and low hypoxemia tolerance. Once our treatment protocol was conducted and OLV with a cuffed SLT was placed on the non-treated side, SpO_2_ remained stable at >90%.

In the AQuIRE registry by Eapen et al., hypoxemia occurred in up to 25% of RB cases, with an increased risk of procedures requiring FiO_2_ < 0.4 to enable thermal ablative procedures [22]. In the patient cohort of Murgu et al., 67.1% of the patients experienced intraoperative hypoxemia and 3.8% major bleeding during RB [24]. As soon as OLV was initiated in our cohort, there were no further cases of hypoxemia. We demonstrated that OLV during RB, even in high-risk patients of ASA classes 3 and 4, prevents hypoxemia and could be a new treatment approach for thermal ablative treatments to safely restore airway lumen patency. No incidence of intraoperative hypoxemia was observed in the three cases, where OLV was used during the entire procedure. 

We report one (4%) case of bronchospasm, no grade 4 bleeding, and one (4%) case of cardiac arrhythmia. Notably, bronchospasm and cardiac arrhythmia occurred during RB before OLV was installed. Although we used OLV in multimorbid patients with reduced physical status and high operative risk, ASA classes 3 and 4, none died within 3 days after the procedure. In total, 79.1% of the patients survived for at least 30 days and reported a subjective increase in their general condition following the endoscopic procedure. Fortin et al. reported a 30-day mortality of 41 (8.1%) patients after RB for malignant airway stenosis with 37 (4.7%) unplanned ICU transfers [28]. The higher rate of ICU admissions in three (12%) patients and the lower 30-day survival in our cohort can be allocated to the higher operative risk of ASA classes 3 and 4 in all patients and advanced cancer stage (AJCC > IIIC) in 54% of the patients. In addition, more than half of our patients had more than four comorbidities.

The primary limitations of the study were its retrospective analysis of a single-center report and the small number of patients. Due to the lack of a control group, safety cannot be compared with patients undergoing RB without OLV. Furthermore, the 30-day mortality is influenced by the multimorbid patient collective, of which 76% are in palliative care. However, the study has several strengths. To our knowledge, no studies detailing a similar procedure can be found in the current literature. This pilot study demonstrates that OLV during RB could be a new treatment approach in patients with lung comorbidities and enables endobronchial ablative treatments requiring FiO_2_ < 0.4, despite intolerance for FiO_2_ < 0.4 being a relative contraindication for endobronchial ablative treatment [2]. Both OLV and RB are established procedures; however, they have not yet been combined in this manner. The only incidence where RB and OLV are used in the same procedure is in pediatrics, where RB is used for placing Fogarty catheters in infants undergoing thoracic surgery to separate both lungs and achieve OLV. This procedure was developed because OLV in infants is a challenging task owing to the narrow airways [29]. Further randomized prospective studies and long-term follow-up are needed to confirm the efficacy and safety of OLV during RB using an SLT.

## 5. Conclusions

OLV using an SLT during RB could be a new treatment approach for endobronchial ablative procedures in patients with pre-existing lung conditions, allowing concurrent high-energy treatments without increasing bronchoscopy-associated risks. Prospective studies are needed to define the role of the OLV with an SLT during RB. 

## Figures and Tables

**Table 1:** Demographic and clinical characteristics.

Demographic Data	Total (*n* = 25)
Male sex	16 (64%)
Age, years	69.16 (± 9.89)
Lung cancer	21 (84%)
Squamous cell carcinoma	9 (36%)
Adenocarcinoma	7 (28%)
Small cell carcinoma	5 (20%)
Lung metastasis	4 (16%)
Cancer location	
Right superior lobe	2 (8%)
Right inferior lobe	2 (8%)
Left superior lobe	2 (8%)
Left inferior lobe	2 (8%)
Perihilar right	12 (48%)
Perihilar left	4 (16%)
Perihilar left and right	1 (4%)
AJCC stages	
IIA	1 (4%)
IIB	1 (4%)
IIIA	2 (8%)
IIIB	1 (4%)
IIIC	1 (4%)
IVA	7 (28%)
IVB	9 (36%)
Comorbidities	
Metabolic comorbidities	
Diabetes mellitus	4 (16%)
Dyslipidemia	4 (16%)
Obesity	3 (12%)
Malnutrition	2 (8%)
Metabolic syndrome	1 (4%)
Heart comorbidities	11 (44%)
Arterial hypertension	10 (40%)
Stroke	3 (12%)
Lung comorbidities	
COPD	10 (40%)
Previous lung embolism	2 (8%)
Asthma	1 (4%)
Previous pneumothorax	1 (4%)
Bronchiectasis	1 (4%)
Smoking	
Active	5 (20%)
Former	16 (64%)
Non-smoker	4 (16%)
Pack years	30 [15–50]
ASA classification	
1	0
2	0
3	18 (72%)
4	7 (28%)
Preoperative mMRC score	
0	1 (4%)
1	3 (12%)
2	6 (24%)
3	7 (28%)
4	8 (32%)

Values are displayed as *n* (%), mean ± standard deviation, or median [interquartile range]. Abbreviations: AJCC, American Joint Commission on Cancer; COPD, chronic obstructive pulmonary disease; ASA, American Society of Anesthesiology; mMRC, Modified Medical Research Council Dyspnea Scale.

**Table 2:** Perioperative procedures.

Perioperative Procedures	
Endobronchial treatment	
Stent	17 (68%)
Laser	16 (64%)
APC	14 (56%)
Forceps	10 (40%)
Electric loop	5 (20%)
Cryoprobe	1 (4%)
RB size before OLV	
8.5	8 (32%)
12.0	9 (36%)
12.5	1 (4%)
14.0	2 (8%)
16.0	1 (4%)
A priori OLV	3 (9%)
Missing data	1 (4%)
RB size during OLV	
6.0	2 (8%)
6.5	7 (28%)
7.0	1 (4%)
7.5	9 (36%)
8.5	4 (16%)
Missing data	2 (8%)

Values are displayed as *n* (%), mean ± standard deviation, or median [interquartile range]. Abbreviations: RB, rigid bronchoscopy; OLV, one-lung ventilation; APC, argon plasma coagulation.

**Table 3:** Perioperative adverse events.

Perioperative Adverse Events	Total (*n* = 25)
Hypoxemia (SpO_2_ < 90%)	22 (88%)
Mean duration (min)	12.5 ± 7.64
Mean lowest SpO_2_ (%)	81.7 ± 6.2
Relevant blood pressure changes	16 (64%)
Increase > 20 mmHg	11 (44%)
Decrease > 20 mmHg	5 (20%)
Bleeding grade 1	2 (8%)
Bleeding grade 2	5 (20%)
Bleeding grade 3	5 (20%)
Bleeding grade 4	0
Procedure-related bronchial fistula	0
Bronchospasm	1 (4%)
New onset abnormal heart rhythm before onset of OLV	1 (4%)
Barotrauma	0
ICU admission	3 (12%)

Values are displayed as *n* (%) or mean ± standard deviation. Abbreviations: SpO_2_, saturation of peripheral oxygen.

## Data Availability

The data that support the findings of this study are available upon reasonable request from the corresponding authors.

## Supplementary Materials

The following supporting information can be downloaded at: https://www.mdpi.com/article/10.3390/jcm12062426/s1, Table S1: Perioperative medications.
