# Supplementary material for: One-Lung Ventilation during Rigid Bronchoscopy Using a Single-Lumen Endotracheal Tube: A Descriptive, Retrospective Single-Center Study
Source: J Clin Med. 2023 Mar 21;12(6):2426. doi: 10.3390/jcm12062426 (PMC10057473; doi:10.3390/jcm12062426)
Supplement: Supplementary file 1 [file jcm-12-02426-s001.zip › jcm-2259518-supplementary.docx]

**Supplementary Material**

**Table S1**. Perioperative medications

| **Medication** |  |
| --- | --- |
| Remifentanil (mcg)  Fentanyl (mcg)  Rocuronium (mg)  Propofol (mg)  Ephedrine (mg)  Noradrenalin (mg) | 957 ± 569  159 ± 76.7  53.6 ± 24.4  607 ± 459  13 ± 11  0.4 ± 0.5 |
